# Applications of Machine Learning in Periodontology and Implantology: A Comprehensive Review

**DOI:** 10.1007/s10439-024-03559-0

**Published:** 2024-06-17

**Authors:** Cristiana Adina Șalgău, Anca Morar, Andrei Daniel Zgarta, Diana-Larisa Ancuța, Alexandros Rădulescu, Ioan Liviu Mitrea, Andrei Ovidiu Tănase

**Affiliations:** 1 University of Agronomic Sciences and Veterinary Medicine of Bucharest, Bucharest, Romania; 2 National University of Science and Technology Politehnica Bucharest, Bucharest, Romania; 3 Minerva University, San Francisco, USA; 4 Cantacuzino National Medical-Military Institute for Research and Development, Bucharest, Romania

**Keywords:** Machine learning, Deep learning, Artificial intelligence, Periodontology, Implant planning, Implant brands and types, Peri-implantitis

## Abstract

Machine learning (ML) has led to significant advances in dentistry, easing the workload of professionals and improving the performance of various medical processes. The fields of periodontology and implantology can profit from these advances for tasks such as determining periodontally compromised teeth, assisting doctors in the implant planning process, determining types of implants, or predicting the occurrence of peri-implantitis. The current paper provides an overview of recent ML techniques applied in periodontology and implantology, aiming to identify popular models for different medical tasks, to assess the impact of the training data on the success of the automatic algorithms and to highlight advantages and disadvantages of various approaches. 48 original research papers, published between 2016 and 2023, were selected and divided into four classes: periodontology, implant planning, implant brands and types, and success of dental implants. These papers were analyzed in terms of aim, technical details, characteristics of training and testing data, results, and medical observations. The purpose of this paper is not to provide an exhaustive survey, but to show representative methods from recent literature that highlight the advantages and disadvantages of various approaches, as well as the potential of applying machine learning in dentistry.

## Introduction

Nowadays, artificial intelligence (AI) is used in numerous domains, such as automotive, robotics, education, or medicine. It mimics the behavior of humans and, most of the time, it leads to faster and better results. A subfield of AI, machine learning covers a plethora of algorithms that learn from data to accomplish various prediction tasks. To better understand the possible medical tasks that can profit from the employment of ML techniques and how the performance of these algorithms can be assessed, an introduction to ML is provided in the current chapter, presenting various types of ML models, existing problems in ML and evaluation metrics. Also, since the object of the current study is the use of ML in periodontology and implantology, a brief description of the two medical areas is provided in the same chapter. The second chapter presents recent surveys and reviews of artificial intelligence techniques in various subfields of dentistry. The third chapter describes the methodology for selecting 48 original papers related to the use of ML algorithms in periodontology and implantology. It also extracts important information from each paper with regards to aim, technical details, characteristics of data, evaluation, and medical observations, trying to identify similar approaches, as well as advantages and disadvantages of various ML techniques. The last chapter draws the conclusions from the analysis of the selected ML solutions.

### Introduction to Machine Learning

A possible classification, based on the learning process [1], groups the machine learning algorithms into supervised ML algorithms (that make predictions based on previously labeled datasets), unsupervised ML algorithms (that process unlabeled datasets and make inferences without the need for human intervention), semi-supervised ML algorithms (that process both labeled and unlabeled data), and reinforcement learning algorithms (that function based on rewards and punishments). Another classification would group ML algorithms based on the task they perform.

#### Types of Machine Learning Algorithms

**Fig. 1:**
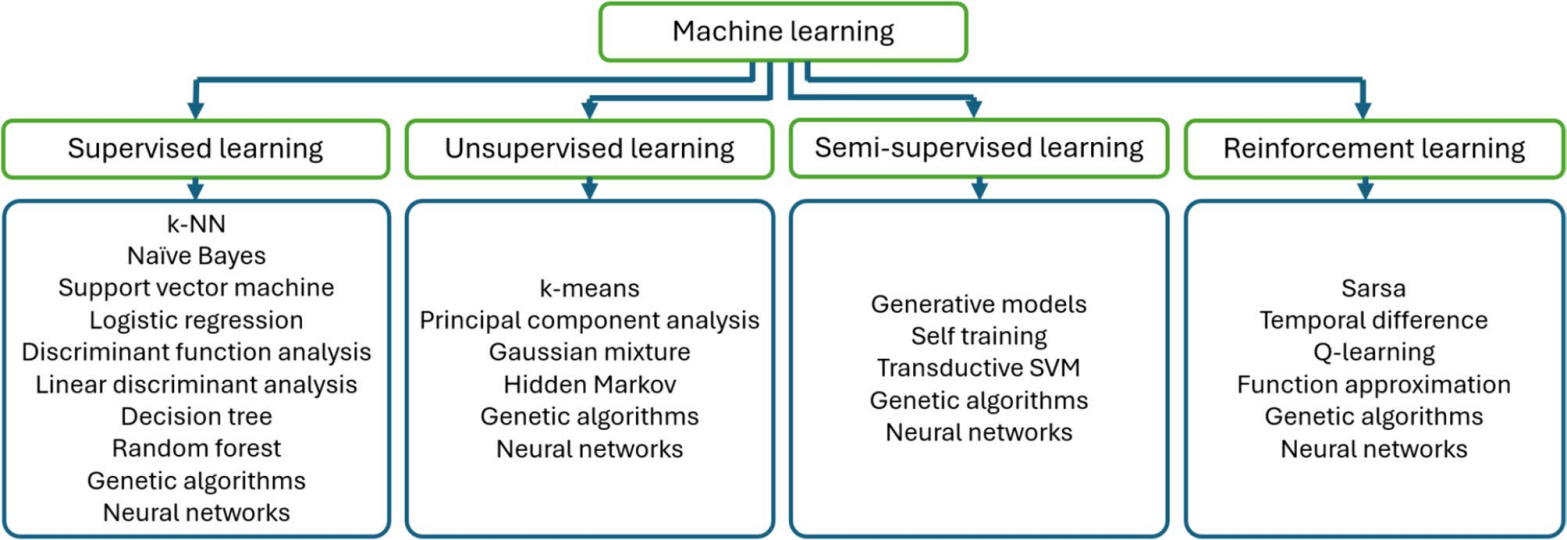
Classification of machine learning algorithms

Figure 1 groups existing ML algorithms according to other proposed classifications [1–4].

While there is a plethora of ML models, as can be observed from Fig. 1, we focus on algorithms applied in dentistry. Many techniques used in periodontology and implantology process radiographic images. Still, there are also several algorithms that process non-clinical data or information that was previously extracted from radiographs (by medical specialists), to deliver a diagnostic or to predict the outcome of a surgical procedure. Most algorithms used in periodontology and implantology belong to the supervised learning category. However, unsupervised learning models can also be applied for segmentation tasks.

K-nearest neighbors (k-NN) [5] is a supervised learning technique that assigns new, unlabeled data, to a category, using training data elements that were previously grouped into certain categories. *Naïve Bayes* is another supervised learning algorithm, based on the Bayes Probability Theorem [6], useful in classification problems. Support vector machine (SVM) [7] algorithms organize data into different categories by using a separation hyperplane that maximizes the differences between classes. Logistic regression (LR) [8] applies a logistic function on a linear combination of independent variables, to predict the result of an output variable. The logistic regression algorithm tries to minimize a cost function that quantifies the error within the predicted probabilities. Discriminant function analysis (DFA) [9] is similar to logistic regression, in the sense that it predicts a categoric result for an output variable, even if the input variables have continuous values. Linear discriminant analysis (LDA) [10] is one of the most popular variants of DFA. It predicts the probability that a certain element belongs to a class or not, using a density function of all the values of the data belonging to that class. Another supervised learning algorithm is a decision tree (DT) [11], which uses a series of rules to classify a new element. The elements in the dataset are organized in a hierarchical structure, where each decision node corresponds to a test that divides the dataset based on the values of a feature, and each leaf node corresponds to a class. To classify a new element, the algorithm starts from the root (the whole, un-labeled dataset), and advances downwards on the nodes that correspond to the values of the element’s features. A random forest (RF) [12] represents a set of decision trees that are obtained from a randomly chosen sub-set of the training dataset. This algorithm combines the votes from several decision trees to assign the new element to a category. Neural networks (NN) are a subset of machine learning models that are inspired by the human brain. Even though neural networks cannot be grouped into a single category based on the learning process, as can be observed in Fig. 1, in the context of periodontology and implantology they depend on previously labeled data, therefore belonging to the supervised learning category. A neural network is comprised of interconnected neurons, organized in three types of layers: input, hidden and output layers. An artificial neural network (ANN) is a neural network that processes the input information only in the forward direction. A recurrent neural network (RNN) is a variant of ANN that contains cycles, so that the output of some neurons can affect the input of the same neurons, in subsequent iterations. A deep neural network (DNN) is a neural network with multiple hidden layers. Convolutional neural networks (CNN) are neural networks specialized in image processing due to the use of kernels that extract salient characteristics from input data with convolution operations. Unlike other neural networks that process previously extracted features from images, CNNs can be applied on raw data. A comprehensive review of artificial neural networks and their applications to computer vision is provided by Abiodun et al. [13].

K-means [14] is one of the simplest unsupervised machine learning algorithms. It divides the elements in a dataset into *k* clusters, based on similarities or dissimilarities. Each element is assigned to a cluster based on a distance function minimization. Genetic algorithms [15] represent computational models of biological evolution. They cannot be grouped into a single category of ML algorithms based on the learning process. However, in the context of medical imaging, they can be employed for clustering tasks (in an unsupervised manner), similar to k-means. They perform natural selection of the fittest individuals by solving an optimization problem. The optimization is accomplished by mixing genetic material from the parents.

#### Tasks in Machine Learning

The classification task refers to assigning a new element into one of several categories, based on the training data which contains previously labeled elements. An example of a classification task is detecting the type/manufacturer of an implant based on a patient’s radiography. This problem can be solved with a multitude of AI techniques, including k-NN, Naïve Bayes, SVM, LR, DFA, decision trees or neural networks.

Unlike the classification task, where the classes are already known, the clustering task refers to identifying similarities between elements in an unlabeled dataset. In computer vision, the clustering problem translates into the segmentation task. An example of segmentation in dentistry is detecting teeth pixels in radiographic images, based on tissue density. A popular algorithm for clustering is k-means. An alternative would be the use of genetic algorithms for energy optimization in active contours-based [16] or in edge-based segmentations [17].

Feature extraction is another task in the ML domain. It is necessary when working with neural networks that cannot process raw data such as full images. Several ML models, such as ResNet [18], or MobileNet [19], can be adapted to extract features (for example, a common feature extraction technique is to use ResNet to process an image and to obtain the output of the network from intermediate layers).

The object detection task assumes the identification of an object’s position and bounding box, as well as its labeling into one of the existing classes. An example of object detection in dentistry would be obtaining a bounding box with a characterization of each tooth (e.g., if it’s healthy or if it has caries) in a radiographic image. Some of the most successful object detection models are you only look once (YOLO) [20], single shot multibox detector (SSD) [21], regions with convolutional neural networks (R-CNN) [22] and region-based fully convolutional networks (R-FCN) [23].

Semantic segmentation is another task in computer vision, where each image pixel is assigned to a class of objects. An example of such task in dentistry is obtaining a mask of pixels for all the implants in a radiography. U-net [24], DeconvNet [25] and SegNet [26] are popular convolutional neural networks, with an encoder-decoder architecture, that perform pixelwise classification.

Instance segmentation is a combination of object detection and semantic segmentation: it performs pixelwise classification, but it also differentiates between distinct objects from the same class (e.g., each mask of pixels belonging to a molar in a radiographic image would have a different label). Some of the most common instance segmentation networks are DeepNet [27] and Mask R-CNN [28].

#### Evaluation Metrics

There are various metrics used to assess the performance of a machine learning algorithm, depending on the problem it tries to solve.

In the binary classification problem, each prediction can lead to one of four results: *true positive* (*TP*—the value was true and it was predicted true), *true negative* (*TN*—the value was false and it was predicted false), *false positive* (*FP*—the value was false, but it was predicted true) and *false negative* (*FN*—the value was true, but it was predicted false). One of the most popular metrics is *accuracy* (*Acc*), measuring the number of correct predictions relative to the total number of input samples. It is defined as:

1 $$Acc= (TP+TN)/(TP+TN+FP+FN)$$

The accuracy is a suitable metric when the target variable classes are relatively balanced (for example, for two classes, the number of items assigned to the first class is approximately the same as the number of items assigned to the second class). Another metric is the *misclassification rate*, or the error rate (*Err*), measuring the number of wrong predictions, relative to the total number of input samples:

2 $$Err=(FP+FN)/(TP+TN+FP+FN)$$

In case of imbalanced classes, more suitable metrics are the precision and the recall. The precision (*P*), or the positive predictive value (*PPV*), refers to the proportion of correct positive predictions. It represents the ratio between true positives and predicted positive values:

3 $$PPV=TP/(TP+FP)$$

The negative predictive value (*NPV*) refers to the proportion of negative predictions. It represents the ratio between true negatives and predicted negative values:

4 $$NPV=TN/(TN+FN)$$

The recall (*R*), also known as the *sensitivity*, or the true positive rate (*TPR*), aims to identify the proportion of actual positive values that were identified incorrectly. It represents the ratio between the true positives and the total number of positive values, either correctly predicted as positives or incorrectly predicted as negatives:

5 $$TPR=TP/(TP+FN)$$

A high value of the TPR is equivalent to a small number of false negatives, whereas a high precision is equivalent to the minimization of the false positives.

*F1 score*, also known as *F-measure* or the *Dice coefficient*, combines the precision and recall metrics, being also very suitable for imbalanced classes:

6 $${F1}=2\cdot P\cdot R/(P+R)$$

Another popular metric is the *specificity*, or the *true negative rate* (*TNR*). It is defined as:

7 $$TNR=TN/(TN+FP)$$

The false positive rate (*FPR*) is defined as:

8 $$FPR=FP/(FP+TN)$$

The receiver operating characteristic (*ROC*) curve shows the performance of a classification model at different discrimination thresholds, by plotting the TPR against the FPR. A popular metric, namely the area under the ROC curve (*AUC*), computes the performance across all the thresholds. A high value of AUC (close to 1) is obtained for correct predictions (close to 100%). The *Youden index (J)*, a common summary measure of the ROC curve, indicates a model’s ability to balance the sensitivity and the specificity:

$$J=TPR+TNR-1$$. (9)

A *confusion matrix* is a tabular representation of prediction outcomes of a classifier (binary or general), illustrating the prediction values and the actual values. The values on the diagonal represent the number of elements for which the predicted label is the same as the actual label (for the binary classification, the diagonal would contain the true positives and the true negatives). The values that are not on the diagonal represent elements that were not correctly labeled by the classifier.

*Cross-validation* is a technique that evaluates the ability of ML models to predict new data that was not used in estimating it. It divides up the training data into k-folds (for example, k = 10). While each time a fold remains out, the model is trained on the remaining data. The model is then used to predict the answers for the observations in the held-out fold. This technique identifies problems such as overfitting or selection bias.

The previously mentioned metrics could be used for classification tasks (for example, to differentiate between various implant brands and types, based on radiographs), as well as for segmentation (e.g., to segment a radiographic image into pixels that belong to healthy teeth, implants, caries, or other tissue).

A very popular metric, used for segmentation or object detection tasks, is the intersection over union (IoU). In the object detection problem, each object has an associated bounding box. The IoU is a measure of the difference between the predicted bounding box and the ground-truth bounding box. It is defined as the ratio between the area of overlap and the area of union for the two bounding boxes. While in the object detection task this measure is computed at bounding box level, in the segmentation task the IoU is computed pixelwise.

Another ML task in dentistry is the estimation of various characteristics in radiographic images, such as root or implant length. A popular metric to assess the quality of an estimator is the mean squared error (MSE), or the mean squared deviation (MSD). It is defined as:

10 $$MSE=\frac{1}{n}\cdot {\sum }_{i=1}^{n}{({x}_{i}-{\widehat{x}}_{i})}^{2}$$

where ${x}_{i}$ represent actual values and ${\widehat{x}}_{i}$, predicted ones. The root-mean-square error (*RMSE*), also known as the root-mean-square deviation (*RMSD*), is defined as the square root of MSE. Low values of MSE and RMSE indicate a good performance of the estimator.

The quality of a classification model or of an estimator can also be accomplished by computing the correlation between the results of an automatic process with those of a manual one. The *Pearson correlation* measures the strength and direction of the relationship between two sets of data. It is a linear correlation, computed as the ratio between the covariance of two variables and the product of their standard deviations. The Pearson correlation has a value in the $\left[-\text{1,1}\right]$ interval, the two extremes corresponding to perfect correlations. A value close to 0 indicates no correlation between the two sets of data. Other statistical comparisons between automatic and manual processes can be performed with the *one-way ANOVA test* [29] or with the *Tukey test* [30], both comparing the means of two or more independent sets of data to determine whether there is statistical evidence that the means of the sets are significantly different.

### Introduction to Periodontology and Implantology

Periodontology is concerned with the study of the tooth’s supportive tissue, with the associated diseases and their treatment. The word “periodontology” has Greek roots. The prefix “peri” means “around,” and “odontos” means tooth. The periodontium refers to the entire area surrounding the tooth. The periodontium is made up of four different types of tissues. On the exterior we have the visible structure, namely gum. Next is the periodontal ligament or desmodontium. This has the role of connecting the tooth to the alveolar bone. The third is radicular cementum, which covers the tooth’s root and fixates the fibers of periodontal ligaments. Finally, we have the alveolar bone which is part of the maxilla bone and contains the root of the tooth, fixating the fibers of periodontal ligaments. The periodontium is of two types: superficial, including gingiva, and deep, including ligaments, cement, and bone [31]. For the periodontium to work within normal parameters and to ensure the health and protection of the tooth, each one of the four tissues must be completely healthy.

When periodontal diseases appear, they tend to first affect the superficial periodontium, i.e., the gingiva. This is called gingivitis. It is characterized by swelling, redness, and bleeding of the gingiva [32]. It is caused by poor oral health and biofilm accumulation [33]. If the biofilm is removed, the gingiva can recover. If the biofilm persists, then gingivitis can lead to affliction of the profound supportive tissues, causing periodontal disease [34, 35]. Untreated periodontal disease leads to irreversible destruction of the profound periodontal tissue and to gingival pockets between the tooth and the supportive bone. When gingival pockets form, clinical signs include dental mobility, secondary migrations of the teeth, gingival recession, bleeding gums, and halitosis. All these factors can lead to loss of teeth [36].

When loss of teeth occurs, due to periodontal disease or other causes, a dental implant is required to accommodate new teeth, so that a patient can use the normal functions of the dental-masticatory complex. Dental implants act as artificial dental roots. With the help of dental prosthetics, dental implants can be a good functional and aesthetic replacement for missing teeth. Dental implants aren’t affected by bacteria itself, but bacteria can affect the structural tissue surrounding the implant, causing peri-implantitis. Peri-implantitis is the equivalent of periodontal disease for natural teeth [37].

When periodontal disease or peri-implantitis appear, there are three types of treatments. The first one is etiologic therapy. It includes patient education for good oral hygiene, removal of biofilm, removal of supra- and subgingival tartar, and treatment of all existing dental problems. Removal of bacteria at home is carried out through a good daily dental brushing, flossing, and waterpik. When needed, the patient is called to the clinic where a more thorough removal of bacteria is carried out by mechanical, airflow, perioflow, laser, sonic or ultrasonic instruments. The second type of treatment is surgical therapy. There are two types of surgical therapy: reducing gingival pockets and correcting the anatomical and morphological defects. Finally, the third type of treatment is maintenance and support of obtained results of the previous treatments, to prevent relapse of the disease [38].

Keeping in mind the importance of detecting and treating dental problems before they can cause further complications, regular check-ups at the dentist are essential. However, detection and treatment can be difficult, even for experienced dental professionals, so any tool that can help the dentist to detect and treat these diseases can have a large positive impact [39]. The onset of AI in the dental sector offers exciting opportunities to assist dental professionals in a variety of ways, ranging from interpreting dental images, correlating risk factors with general health problems, detecting type of implants and many other benefits.

## Related Work

In recent years artificial intelligence has led to the advance of many areas in medicine, including dentistry. While several surveys give an overview of the whole field of dentistry [40–44], others focus on selected areas, such as implantology or periodontology [45–52].

General surveys provide overviews of various sub-domains of dentistry. Shan et al. [40] outlined the progress and the potential applications of AI in dentistry, which range from diagnosis and treatment to disease prediction. Katne et al. [41] presented applications of AI in various fields of dentistry, including general dentistry, oral and maxillofacial surgery, oral medicine, dental and maxillofacial radiology, forensic odontology, dental education system, prosthodontics, orthodontics, and periodontics. Grischke et al. [42] provided an overview of existing applications of robotic systems and AI in dentistry. They mentioned several robotic assistants in dental implantology, and machine learning tools to predict periodontal diseases or peri-implant infection. However, they pointed out that the use of AI is still restricted to pilot use cases and narrowly defined research questions. Schwendicke et al. [43] identified opportunities and challenges of AI-based dental diagnostics and treatment planning. Kang et al. [44] provided an analysis of deep learning methods used in dentistry and implantology. They identified applications for image quality enhancement, detecting teeth or dental caries, diagnosing periodontal diseases or cancerous lesions, identifying cephalometric landmarks, and assisting during the manufacturing of prostheses.

More focused reviews analyze the use of ANNs or CNNs in periodontology and implantology. Bernauer et al. [48] aimed to assess the usefulness of using ANNs or CNNs in several dentistry tasks, such as identifying and classifying dental implant systems, assisting in the fabrication of implant-supported monolithic zirconia crowns cemented on customized hybrid abutments, predicting periodontally compromised teeth, or classifying teeth in dental prosthetics workflows. Manerikar et al. [50] identified several applications of CNN in different sub-areas of periodontology and implantology, such as dental plaque detection, identification of gingivitis, detection of periodontal diseases, as well as classification of implant design systems. Revilla-León et al. [51] provided a survey of AI models for detecting dental plaque and for diagnosing gingivitis and periodontal disease. The performance evaluation indicated that AI models for detecting plaque (from 2 studies) obtained an accuracy ranging from 73.6 to 99%, while solutions for diagnosing gingivitis (8 studies) reached an accuracy ranging from 74 to 78.2% for intraoral photographs and from 67.7 to 73.72% for fluorescent intraoral images. Three research papers included in their study, related to the diagnosis of periodontal disease, reported an accuracy between 47 and 81%. Analyzing the performance of AI models for detecting alveolar bone loss on 11 studies, they observed an accuracy ranging from 73.4 to 99%. Mohammad-Rahimi et al. [52] included 47 studies in a review of deep learning solutions in periodontology and oral implantology. Their uses cases included the detection of periodontitis and gingivitis or periodontal bone loss, the classification of dental implant systems and the prediction of treatment outcomes in periodontology and implantology.

Other surveys focus on the use of AI in various tasks of implantology such as implant type recognition, implant success prediction or customization of prostheses. Revilla-León et al. [45] provided an analysis of AI models in implant dentistry for implant type recognition (7 studies), implant success prediction (7 studies) and implant design optimization (3 studies). Even though they reviewed a small number of research papers, their conclusions regarding the performance of AI models (accuracy between 93.8 and 98% for solutions that recognize implant types, and between 62.4 and 80.5% for methods that predict osteointegration success) are very encouraging. Saghiri et al. [46] analyzed 10 research papers published between 2000 and 2020 that are related to technology used in the identification of different implant systems. Another recent study [47] reviewed 4 pre-trained CNNs used for the identification of dental implant systems. Out of the selected articles, they extracted information about implant systems, imaging modality, training sample size, validation method, AI architecture and evaluation metrics. The accuracy of the studied AI models ranged from 51 to 99.5%. Pareek and Kaushik [49] provided another review that focuses on recent AI models in dental prosthetics and their efficacy in diagnosing and building customized prostheses.

Among the limitations identified in various surveys we mention the dependence on datasets and the lack of awareness in follow-up treatment [49]. Common limitations of CNN-based applications in periodontology are related to the input data (sample size, image resolution) and to the use of 2D periapical radiographs, as opposed to computer tomography (CT) or magnetic resonance imaging (MRI) datasets [50]. Schwendicke et al. [43] identified a possible future scenario that would considerably improve the field of dentistry, where the training sample size shifts from several thousands to millions of multi-level connected instances, the focus moves from the detection of structures on imagery, association modelling, to multi-class detection of pathologies, predictive modelling, and decision support. They also foresaw a progress, where the testing mode would change from cross-validation to hold-out test sets and independent datasets and where evaluation metrics would not target only measures of accuracy (e.g., accuracy, sensitivity, specificity, area under the curve, F1-score, etc.), but also measures of value (impact on treatment decision, cost-effectiveness) and trustworthiness of AI (explainable AI).

While several surveys are too general, analyzing the use of AI models for all the sub-areas of dentistry, others focus only on specific AI techniques (for example, only deep learning methods) in periodontology and/or prosthodontics, including a very small number of research works in their studies. The purpose of the current research is to provide an overview of all types of ML techniques applied in periodontology and implantology, to identify common procedures based on use cases, to highlight out strengths and weaknesses of various solutions and to point out interesting medical observations.

## Selected Papers

### Selection Procedure

We used several scientific databases to search for recent applications of artificial intelligence in periodontology and implantology (between 2016 and 2023): PubMed, IEEE Xplore, ScienceDirect and Google Scholar. The search keywords included terms from both the AI domain (“artificial intelligence” or “machine learning” or “deep learning”) and from the targeted sub-areas of dentistry (“peri-implantitis” or “periodontology” or “implant planning” or “implantology” or “implant”). The resulting research papers were first screened based on title and abstract and the remaining items were evaluated based on full-text reads, resulting in an initial group of approximately 40 papers. The second selection step led to an additional group of roughly 20 papers, by cross-referencing the initial group. Out of the approximately 60 papers, the surveys and reviews were analyzed separately in the Related work section. We reached a total of 48 original research papers that were included in the current study.

### Proposed Classification

The original papers were classified into four categories: periodontology (*n = *11), implant planning (*n = *9), implant brands and types (*n = *14) and success of dental implants (*n = *14).

### Periodontology

The *periodontology* category consists of scientific works aimed at predicting teeth in need of extraction or that are periodontally compromised, detecting periodontal bone loss (PBL) or staging periodontitis.

Lee et al. [53] aimed to develop a CNN model for the diagnosis and prediction of periodontally compromised teeth. They applied a deep CNN architecture, using periapical radiographic images. Kim et al. [54] used deep CNNs with transfer learning and clinical prior knowledge, to detect PBL. They trained U-shaped networks to extract regions of interest containing the teeth, as well as a multi-label classification network that predicts the existence of PBL in each tooth. Krois et al. [55] applied a deep feed-forward CNN to detect PBL on panoramic radiographs. Their network was trained and validated using 10-times repeated group shuffling. Also, hyperparameters were systematically tuned using grid search [56]. Comparing the performance of a CNN with that of 6 dentists, the CNN obtained slightly higher accuracy. Thanathornwong and Suebnukarn [57] aimed to adapt the Faster R-CNN [58] model from the natural image domain using a small annotated clinical data, to identify bounding boxes of periodontally compromised teeth. Chang et al. [59] proposed a hybrid method, combining a deep learning architecture with conventional processing for classification, to detect and classify PBL in each individual tooth.

A CNN was employed for the detection of the periodontal bone level, the cementoenamel junction (CEJ) level, and the individual teeth. Next, the percentage rate analysis of the PBL was accomplished based on the tooth long-axis and the periodontal bone and CEJ levels, using the criteria proposed at the 2017 World Workshop on the Classification of Periodontal and Peri-Implant Diseases and Conditions [60]. Lee et al. [61] integrated 3 deep segmentation networks (U-net with ResNet-34/CNN Encoder) for bone area, tooth, and CEJ, and performed measurements of the bone level, assigning the stage of radiographic bone loss (RBL) for each tooth. Kabir et al. [62] applied an end-to-end deep learning network which combines a set of segmentation networks and a classification network to output bone area, tooth and CEJ line masks. The RBL stage assignment follows the periodontitis classification presented by Tonetti et al. [60]. Jiang et al. [63] used a two-stage deep learning architecture based on U-net and YOLO-v4 to localize teeth and key points which led to the calculation of the percentage of bone loss and the staging of the periodontitis. Karacaoglu et al. [64] extracted first-order statistics, shape and size-based features and textual features from periapical images and applied several classifiers (k-NN, SVM, eXtreme Gradient Boosting (XGBoost), RF, LR and DT) on a reduced set of features, to classify periodontal defects.

Unlike other works that handle periodontitis, Deng et al. [65] developed a screening tool for periodontal health status based on non-clinical parameters and salivary biomarkers, using LR and RF on data such as gum disease, rating of gum/teeth health, tooth cleaning, loose teeth, or gingival bleeding on brushing (GBoB). Another research that does not feed radiographic images to the ML models was described by Lakshmi and Dheeba [66]. They applied various classifiers on data containing demographic information, clinical and radiological findings to predict the progression of periodontitis.

Table 1 presents the aim, as well as technical details, characteristics of training/validation/testing data, results of experiments and medical observations of analyzed papers in the periodontology category.

**Table 1:** Information extracted from the selected scientific papers in the *periodontology* category

References	Year	Aim	Technical details	Data	Results	Medical observations
[53]	2018	- Develop a diagnostic and a prognosis of periodontally affected teeth	- A CNN architecture, with 16 convolutional layers, max pooling layers and 3 fully connected dense layers - The training network weights were learned using the Adam algorithm [67]	- Periapical radiographs: 1392 images annotated by 3 periodontists + augmentation - 75% for training - 25% for testing	- *Acc* for periodontally compromised teeth: 0.81/0.767 for premolars/molars - *Acc* for predicting extraction: 0.828/0.734 for premolars/molars	–
[54]	2019	- Detect PBL using DeNTNet (deep neural transfer network)	- U-Net model for teeth extraction - The same U-shape architecture to predict PBL lesions - A multi-label classification network to predict the existence of PBL in each tooth.	- Panoramic dental radiographs: 12179 images annotated by dental clinicians - Testing sets were obtained selecting 11, 189, 190 and 800 images from the initial dataset.	- DeNTNet: *F1-score* of 0.75 - The avg. performance of clinicians was 0.69	- Radiological exams like bitewing, retroalveolar and panoramic radiographs are the most common way to identify periodontitis.
[55]	2019	- Detect PBL	- A deep feed-forward CNN - Grid search for tuning model architectures and hyperparameters - Final model: a 7-layer deep network parametrized by 4299651 weights	- Panoramic dental radiographs: 1750 segments from 85 radiographs + augmentation - The dataset was repeatedly split (10 times) into training (1400) and validation (350) sets with group shuffling.	- The reference test was the maximal detectable PBL in percentage of the tooth length. - CNN: *Acc* 0.81, *Sensitivity* 0.81, *Specificity* 0.81. - dentists: *Acc* 0.76, *Sensitivity* 0.92, *Specificity* 0.63	- Molars had the highest level of accuracy when detecting PBL (for CNN and dental experts) - for incisors, the performance decreased.
[57]	2020	- Identify periodontally compromised teeth using Faster R-CNN	- A Faster R-CNN, with a pretrained ResNet architecture, adapted from the natural image domain	- Panoramic radiographs, annotated by experts in periodontology: 100 images + augmentation. - Training - in a minibatch manner - 70% for training - 10% for validation - 20% for testing	Object detection task: - *Avg. Precision (AP) rate* 0.81 - *Avg. Recall (AR) rate* 0.80 Classification task: - *Avg. Sensitivity* 0.84 - *Avg. Specificity* 0.88 - *F-measure* 0.81	- Timely periodontal disease detection can prevent teeth loss and heart diseases that are associated with periodontitis
[59]	2020	- Detect and classify periodontal disease and PBL	- A modified Mask R-CNN based on feature pyramid network and a ResNet101 backbone – to detect PBL, CEJ level and the teeth	- Panoramic radiographs: 330 images to detect PBL, 115 images to detect CEJ level and 73 images to detect teeth + augmentation - 90% for training - 10% for testing	- *Pearson correlation coef.* of the automatic method with manual diagnoses: 0.73 overall - interclass correlation value 0.91 overall for the whole jaw	–
[61]	2021	- Measure alveolar bone - Evaluate periodontal diseases	- U-net with ResNet-34 for bone area segmentation - U-net with ResNet-34 for tooth segmentation - U-net with CNN encoder for CEJ line segmentation	- Periapical radiographs: 693 images from 37 patients, annotated by 3 specialists - 70% for training - 10% for validation - 20% for testing	- *Dice coef.* 0.91 for segmentation *Acc* - *AUC* of RBL stage assignment for stage I/ II/III: 0.89/0.9/0.9 - *Acc* of case diagnosis 0.85	–
[62]	2021	- Develop a diagnostic, prevention, and treatment plan with HYNETS (hybrid network for periodontitis stages from radiograph)	- A set of segmentation networks that outputs bone area, tooth and CEJ line masks - A classification network	- Periapical radiographs: 700 images annotated by 3 specialists - 70% for training - 10% for validation - 20% for testing	- *Avg. Dice coef.* 0.96/0.94 for bone area/tooth segmentation - *Avg. AUC* 0.97 for periodontitis stage assignment	- CNN can assist clinicians throughout the whole process, including a treatment plan
[63]	2022	- Detect periodontal alveolar bone with YOLO	- U-net for tooth segmentation - CSPDarkNet [68] + spatial pyramid pooling module + path aggregation network + YOLO to generate class probabilities, object scores and bounding boxes	- Panoramic radiographs: 640 images annotated by 3 experts + augmentation - 80% for training - 20% for testing	- Classification *Acc* 0.77	- The classification accuracy of the deep learning model is higher than that of general practitioners
[64]	2023	- Classify periodontal defects on periapical images	- Feature extraction: 1409 quantitative imaging features which were grouped in three classes (first-order statistics, shape and size-based features, textual features) - SelectKBest methos, least absolute shrinkage and selection operator (LASSO) – employed for feature reduction (resulting in 4 features) - 6 classifiers: k-NN, SVM, XGBoost, RF, LR and DT	- 87 periapical images - 80% for training - 20% for testing	- Best results: SVM - For no periodontal defects - *Precision* 0.8 - *Recall* 1 - *F1-score* 0.89 - *AUC* 0.75 - For periodontal defects - *Precision* 0.2 - *Recall* 1 - *F1-score* 0.33 - *AUC* 0.75	- They claim that diagnostic information by periapical and bitewing projections is the most valuable when evaluating periodontal disease
[65]	2023	- Develop a screening tool for periodontal health status based on non-clinical parameters and salivary biomarkers	- LR and RF to classify periodontal health level	- 408 patients: both healthy people and patients with various stages of periodontitis - data: gum disease, rating of gum/teeth health, tooth cleaning, loose teeth, use of floss, aMMP-8 POCT, GBoB, hemoglobin, age	- RF: *AUC* 0.94 for the discrimination of three classes (health, gingivitis, periodontitis) and 6 classes (health, gingivitis, stages I, II, III, IV periodontitis)	- Screening of periodontal health status in non-clinical settings may provide utility to manage preventive and therapeutic services/care pathways
[66]	2023	- Predict the progression of periodontitis	- NB, SVM, RF, LR, k-NN, DT classifiers	- 1000 periodontitis patients (no images are fed to the ML): 493 with chronic generalized periodontitis and 507 with chronic localized periodontitis - Features: demographics (age, gender, smoking use, etc.), clinical findings (gingival index, tooth mobility, etc.), radiological findings - 80% for training - 20% for testing	- *Acc*: NB 0.955, SVM 1, RF 1, k-NN 0.995, LR 1, DT 0.99	- Periodontitis cannot be healed but may be managed - Early stage of gingivitis can be cured with correct prompt treatment and oral hygiene

Artificial intelligence fills the need to identify, measure, classify, put a diagnostic and prognosis on periodontal disease more efficiently. The most common imaging modalities for periodontology are bitewing, retroalveolar, and panoramic radiographs. As can be seen from Table 1, the most popular ML algorithms in periodontology are CNNs, which are capable of segmenting teeth and CEJ lines, and predicting PBL lesions, based on radiographic images. Other ML classifiers, such as NB, SVM, RF, LR, k-NN, or DT, process data that was previously extracted from radiographs by medical experts, or non-clinical data (e.g., age, gender, smoking use, etc.).

From the analyzed papers in this category, it can be observed that the training datasets are relatively small (ranging from 100 to 1750 in 7 of the 8 papers, and only one paper with 12179 radiographs). However, data augmentation can compensate for the small sample size. Regarding the evaluation of the proposed solutions, the papers assess the success of different tasks in periodontology: detection of periodontally compromised teeth, prediction of extraction, detection of PBL, staging of periodontitis, segmentation of teeth and CEJ lines. Also, each paper uses different measures: either accuracy, F1-score/F-measure/Dice coefficient, recall/sensitivity, specificity, precision, or AUC. In any case, the evaluation results show that AI is on par or even surpasses the ability of general practitioners to classify periodontal disease. However, automatic solutions are by no means a replacement for medical practitioners, but rather an enhancement to their ability to identify and combat periodontal disease. Some papers hint to the fact that AI models and medical practitioners alike should correlate radiological images with patient dental status.

With the help of AI, dental practitioners can identify periodontal disease faster, thus lowering the chance of tooth loss, cardiovascular disease and other negative health effects associated with periodontal disease.

### Implant Planning

The papers in the *implant planning* category are aimed at detecting regions with missing teeth (and their properties), at determining the implant length and the cervical width, and at predicting the location for the insertion of implants.

Görler and Akkoyun [69] studied the potential of using a layered feed forward ANN to efficiently determine canine implant length and cervical width from panoramic radiographs. Lee et al. [70] applied a CNN with multi-phase training and preprocessing on cone-beam computed tomography (CBCT) images for tooth segmentation (in the context of implant planning), using volumes of different sizes. Roongruangsilp and Khongkhunthian [71] aimed to investigate the learning curve of AI for dental implant treatment planning in the posterior maxillary region. They compared the learning curves of the Faster R-CNN algorithm (using the IBM PowerAI Vision platform) in determining areas with missing teeth and implant size, using four data augmentation procedures: blur, sharpen, color and noise. Bayrakdar et al. [72] applied a CNN to detect bone height and thickness, canals/sinuses/fossae in missing tooth regions. Park et al. [73] aimed to improve the implant planning process by using Mask R-CNN with ResNet-101 for the tooth segmentation task and Faster R-CNN with ResNet-101 for predicting regions of missing teeth. Moufti et al. [74] developed a U-net CNN to segment edentulous alveolar bone (area lacking teeth) in CBCT images in the implant planning phase. Oliveira-Santos et al. [75] proposed a solution that detects the mandibular canal (MC) even in the presence of anatomical variations such as the anterior loop (AL).

Hashem et al. [76] proposed an automatic solution that predicts the exact location of the implant with Guided Local Search with Continuous Time Neural Network (GLCTNN). Liu et al. [77] explored the capability of an AI system in automatically designing implant planning (predicting the implant location). They used Single Shot Detector (SSD) and V2V-PoseNet for edentulous site and related key points detection, for generation of implant axis and computation of implant position.

Table 2 presents the aims, the technical details, information about the data used for training/validation/testing, the results of the evaluation and several medical observations in the *implant planning* category.

**Table 2:** Information extracted from the selected scientific papers in the *implant planning* category

References	Year	Aim	Technical details	Data	Results	Medical observations
[69]	2017	- Determine canine implant length and cervical width	- An ANN to approximate tooth root size - Neurons in the hidden layer have tangent hyperbolic function - Neurons in the output layer have linear activation function	- Panoramic radiographs: 120 images with normal canine teeth - 80% for training - 20% for testing	- *MSE* 0.34 mm for root length (error values 2%) and 0.29 for cervical width (error values 4.4%)	- Failure of implants can be due to biological factors, unsuccessful osseointegration, periimplantitis or complications involving a fracture
[70]	2020	- Tooth segmentation for implant planning	- A CNN based on the U-net architecture - The convolutional layers were replaced with dense convolution blocks	- CBCT images + augmentation - 69 datasets with 1066 images for training - 1 dataset with 400 images for validation - 32 datasets with 151 images for testing	- Comparison with a level-set based method - *Dice value* 0.82 for validation set and 0.79 for test set (level-set based method) - *Dice value* approx. 0.95 for the proposed method	- Advantages of CBCT: can be used as a guide for implant planning (shows angulation, dimension, and diameter of implant)
[76]	2020	- Predict the exact location for insertion of implants on jawbone	- GLCTNN to predict the implantation location	- Dataset from [78]	- *Avg. Acc* 0.995 – the best, compared to W-J48 [79], Naïve Bayes, SVM, K-NN [80], Nearest Neighbors with Structural Risk Minimization (NNSRM) [81] and Generalized Regression Neural Network (GRNN) [82]	- Implant inserting by dental professionals is not fully controllable and it demands costly and time-consuming training
[77]	2021	- Design automatic implant plan (implant position)	- SSD to detect missing tooth position - Bone contour is detected, and the designed characteristics annotators are captured with V2V-PoseNet - Implant axis and implant position are computed	- CBCT images: 2500 images to train and develop the model parameters - 67 cases to determine the hyper parameters - 12 patients with missing left molars, divided into 3 groups	- Comparison between automatic position estimation and actual position - Statistical comparison using *One-way ANOVA* and *Tukey’s test* - Best results: coronal dev. 0.6638 ± 0.2651 mm, apical dev. 1.157 ± 0.3350 mm, angular dev. 5.307 ± 2.891°	- Repositioning the dental implant radiography in a 3D fashion can reduce human error and can help with guided surgery - The sample size of this study may be insufficient
[71]	2021	- Investigate the learning curve of AI in implant planning (determining areas with missing teeth and implant diameter and length)	- A Faster R-CNN algorithm - They compared 4 data augmentation procedures (blur, sharpen, color, noise)	- CBCT images: 184 datasets - 316 implant position images: 6 random datasets (each with 50 images) for training, 16 images for testing	- Blurred augmented models improved *detection* by 12.5%, but showed less *accuracy* than the original model by 18.34% - The learning curves of the other 3 augmented models (sharpen, color, noise) increased in both *detection* and *accuracy*	- Based on bone quality, patient evaluation and a good treatment plan lead to a good chance of long term success
[72]	2021	- Detect bone height and thickness, canals/ sinuses/ fossae in missing tooth regions	- They used Diagnocat, a deep CNN based on a fully modified 3D U-net architecture [83]	- CBCT images: 75 images annotated (used only for testing) - Annotated elements: bone height and thickness, canals/sinuses/fossae associated with alveolar bone missing tooth regions	- Percentage of correct *detection*: 72.6% for canals, 66.4% for sinuses/fossae and 95.3% for missing tooth regions	- No significant differences in measurements taken by humans and computed with AI (except in bone thickness)
[73]	2022	- Detect regions with missing teeth for implant planning	- Mask R-CNN [28] with ResNet-101 [18] as backbone to segment teeth - Faster R-CNN with ResNet-101 backbone to predict regions of missing teeth (trained on synthetic dataset)	- Panoramic radiographs: 455 images + augmentation - Dataset split into 77.5% for training, 7.5% for validating and 15% for testing - Synthetic dataset (images with healthy teeth, randomly removing teeth based on tooth instance segmentation)	- *Mean Avg. Precision* (*mAP*) 92.14% for tooth segmentation - *mAP 59.09*% for missing tooth regions detection	- CBCT images are desired, as compared to panoramic radiographs, for dental implant placement
[74]	2023	- Segment the edentulous mandibular bone for implant planning	- A U-net CNN (from MONAI framework [84]) that was trained, under supervision, from scratch	- CBCT images: 42 labeled cases - 33 for training - 10 for testing	- *Dice coef.*: 0.89 for training and 0.78 for testing - Unilateral areas: *Dice coef.* 0.91 - Bilateral cases: *Dice coef.* 0.73	- Very small sample size - The current model is limited to the mandible and to the bounded saddles
[75]	2023	- Detect MC for implant planning	- A 3D U-net architecture: an encoder-decoder fully convolutional network with skip connections	- CBCT images - 40 CBCT scans for the development of the AI network - 219 CBCT scans for training (126 for initial training and 93 for training of the optimization of the AI to detect the AL) - 27 for validation - 27 for testing	- Detection of MC with AL: *IoU* 0.659, *Dice coef.* 0.792, *Precision* 0.677, *Recall* 0.961, *Acc* 0.998 - Detection of MC without AL: *IoU* 0.654, *Dice coef.* 0.789, *Precision* 0.668, *Recall* 0.970, *Acc* 0.997	- Anatomical variations of the MC, such as AL, must be detected to avoid nerve injury during surgeries

Artificial intelligence in implant planning encompasses key applications like tooth segmentation refinement and automated implant plan design. As can be observed from Table 2, the chosen ML algorithms in implant planning are usually ANNs or CNNs: U-net or Mask R-CNN for pixel-based tooth segmentation, variations of R-CNN (e.g., Faster R-CNN) or SSD for detection of missing teeth’s bounding boxes, ANN to approximate tooth root size, etc. Even if their selected ML model was still a neural network, Hashem et al. [76] also compared the performance of their solution with that of other ML algorithms (W-J48, Naïve Bayes, SVM, K-NN, NNSRM, and GRNN), obtaining the best results in terms of accuracy.

In some scientific works panoramic radiographs are used to detect regions with missing teeth or implant characteristics. However, CBCT images are desired, since they provide a 3D, more exact guidance regarding the position of the implant. The sample sizes in the selected papers range from 42 images to 2500 images, with different proportions for training, validation, and testing. Even if the results hint to the potential of AI, the sample sizes may be too small for a thorough evaluation of the proposed solutions.

Notable achievements include improved tooth segmentation via modified CNNs and successful AI-driven predictions of implant positions, highlighting AI's potential to minimize errors and support dental decision-making, ultimately improving patient evaluation and treatment plan in order to achieve long-term success of the implant.

### Implant Brands and Types

The solutions in the *implant brands and types* category aim to classify brands and models of implants. Most of the selected papers [85–94] evaluate the efficacy of CNNs in identifying models of implants in radiographic images, either panoramic or intraoral (periapical). Other scientific works [95, 96] compare the performance of clinicians and that of deep learning models in classifying implants. Benakatti et al. [97] analyze the performance of SVM, LR, k-NN and X boost classifiers in identifying implant types based on shape, using Hu and Eigen values.

Table 3 summarizes the technical details, the information about the dataset used for training/validation/testing, the results of the evaluation and several medical observations.

**Table 3:** Information extracted from the selected scientific papers in the *implant brands and types* category

References	Year	Aim	Technical details	Data	Results	Medical observations
[85]	2020	- Classify types of implants brands	5 different CNNs: - Basic CNN with 6 convolutional layers - Transfer-learning VGG16 / VGG19 with pre-trained weights - Transfer-learning and fine-tuning VGG16 / VGG19	- Panoramic radiographs: 8859 images of 11 implant systems - 75% for training - 25% for testing	*Accuracy* - Basic CNN: 0.86 - VGG16-transfer: 0.9 - VGG16-fine tuning: 0.94 - VGG19-transfer: 0.88 - VGG19-fine tuning: 0.93	–
[86]	2020	- Identify the brand and model of implants	- Pretrained GoogleNet Inception CNN to eliminate non-exploitable data - Network architecture with 27 layers, trained using transfer learning	- Intraoral radiographs: 1206 images with 6 models of implants from 3 brands - 80% for training - 20% for testing	- *Acc* 0.938 - *Sensitivity* 0.935 - *Specificity* 0.942 - *PPV* 0.92 - *NPV* 0.915	- The traceability of dental implant models is very important
[87]	2020	- Identify and classify dental implants	- A pretrained model of ImageNet used for preprocessing and transfer learning - GoogleNet Inception-v3 used for classification	- Panoramic radiographs (5390) and periapical radiographs (5380) - 80% for training - 20% for testing	- CNN architecture: *AUC* 0.971 - Periodontists: *AUC* 0.925	- Straumann BLT implant system appears to be the easiest to recognize in radiographic images
[88]	2020	- Identify implant type	- YOLO v3 with fine-tuning - Training dataset separated into 16 batches for every epoch - 1000 epochs with a learning rate of 0.01	- Panoramic radiographs (1282) with 6 types of implant systems from 3 brands - 80% for training - 20% for testing	Classification: - *TP ratio*: from 0.5 to 0.82 - *AP*: from 0.51 to 0.85 Object detection: - *mAP 0.71* - *mIoU* 0.72	–
[95]	2020	- Compare performance of CNN and dental experts	- Automated deep convolutional neural networks (18 layers with no dropout) with Neuro-T version 2.0.1 - Adam optimizer with L2 regularization for transfer learning	- Panoramic and periapical radiographs (11980) with 6 types of dental implant systems - 80% for training - 20% for testing	DCNN: - *AUC* 0.954 - *Youden index* 0.808 - *Sensitivity* 0.955 - *Specificity* 0.853 Dental professionals: - *AUC* between 0.53 and 0.93	- Straumann BLT is easy to recognize by DCNN (AUC 0.98) and dental professionals (0.93) - Osstem TSIII is difficult to recognize by dental professionals (0.54 / 0.53)
[89]	2021	- Identify dental implant type in periapical radiographs with AI	- A CNN architecture with 5 convolutional layers, a pooling layer + 5 dense layers - Categorical Crossentropy loss function and Stochastic Gradient Descent optimization	- Periapical radiographs: 1800 images with implants from 3 manufacturers + augmentation - 80% for training - 20% for testing	- *Acc* 0.85 - *Sensitivity* 0.9 - *Specificity* 0.82 - *PPV* 0.83 - *NPV* 0.89	- Implant type identification is a major challenge before prosthetic restoration of implants (if there is no patient history)
[90]	2021	- Compare performance of 5 CNNs for implant brand and treatment stage classification	- ResNet18, 34, 50, 101 and 152 - They evaluated the multi-task and single-task accuracies	- Panoramic radiographs: 9767 images with 12 implant brands and treatment stages - 80% for training - 20% for testing	- Single-task (for implant brands): *Acc* 0.978-0.985, *Recall* 0.972-0.98 - Multi-task: *Acc* 0.98-0.99, *Recall* 0.972-0.988	- 3% of inserted dental implants end up being removed due to difficulty in identifying the type of implant
[97]	2021	- Evaluate performance of machine learning algorithms to identify implant types	- Algorithms: SVM, LR, k-NN, X boost classifiers - Classify implant types based on shape, using Hu and Eigen values	- Panoramic radiographs of 3 dental implant systems (actual number of images not mentioned) - 80% for training - 20% for testing	- SVM: *Acc* 0.47 (Hu), 0.67 (Eigen) - k-NN: *Acc* 0.33 (Hu), 0.17 (Eigen) - LR: *Acc* 0.5 (Hu), 0.67 (Eigen) - X boost: *Acc* 0.33 (Hu), 0.67 (Eigen)	Limitations: - Images were taken from a single source - A small dataset was used
[96]	2022	- Compare performance of clinicians vs AI in classifying implants	- The Neuro-T version 2.0.1 automated deep learning algorithm was used	- Panoramic images - 5716 implants for training and 1429 for validation – from a previous study - 180 images for testing – annotated by 5 periodontists, 8 periodontology residents and 31 dentists	- Only human classification: *mean Acc* 0.63 - Only AI classification: *mean Acc* 0.81 - Dental professionals assisted by AI: *mean Acc* 0.79	- AI was especially beneficial for unexperienced dentists and elevated their performance close to that of seasoned professionals.
[91]	2022	- Classify implant fixtures with CNN	- YOLO v3, pretrained with a large image dataset of objects - Trained to classify fixtures through transfer learning	- Periapical images: 355 implant fixtures from 3 different systems - 80% for training - 20% for testing	- Best results: network trained for 200 epochs - *Sensitivity* 0.94 - *Acc* 0.97 - Confidence score 0.75	- It is very important to know the dental implant type - Periapical images are more suitable than panoramic radiographs
[92]	2023	- Evaluate the performance of ML for the classification and identification of dental implants	- YOLO v5 and v7 were used to train the model - Real-enhanced super-resolution GAN	- Panoramic radiographs: 14037 implants, divided into 10 classes - 103 types of implants - Data augmentation - 80% for training - 20% for testing - 3-fold cross-validation (implant datasets 1,2 and 3)	- *mAP* of YOLOv7 in the 3 datasets: 0.931, 0.984, 9.884 (higher than *mAP* of YOLOv5) - After image processing in dataset 1: *mAP 0.986* - with real-enhanced super-resolution GAN: *mAP 0.988* (magnification x2) and 0.986 (magnification x4)	- Accuracy of ML model decreases when the number of types of implants increases
[93]	2023	- Evaluate the accuracy of ML for identifying and classifying types of dental implants	- The Neuro-T version 3.0.1 automated deep learning algorithm was adopted in this study	- 156965 panoramic (116756) and periapical (40209) radiographic images - 10 manufacturers - 27 types of implants	- Using both panoramic and periapical images: *Acc* 0.8853, *Precision* 0.857, *Recall* 0.823, *F1-score* 0.84 - Using only panoramic images: *Acc* 0.8789, *Precision* 0.852, *Recall* 0.811, *F1-score* 0.831 - Using only periapical images: *Acc* 0.8687, *Precision* 0.844, *Recall* 0.817, *F1-score* 0.83	- No statistically significant difference in accuracy performance between panoramic and periapical images - Highest accuracies for Nobel Biocare Branemark, Megagen Exfeel external, Osstem US III, Dentsply Xive implants - Lowest accuracies for Warantec IT implants (due to small number of images)
[94]	2023	- Classify dental implant systems using cloud-based deep learning algorithm	- Google automated machine learning (AutoML) Vision – executed a neural architecture search to apply an appropriate algorithm - A single-label image classification model was trained using AutoML	- Periapical radiographs: 4800 (1200 for each of the 4 implant systems) - 80% for training - 10% for validation - 10% for testing	- *Acc* 0.981, *Precision* 0.963, *Recall* 0.961, *Specificity* 0.985, *F1-score* 0.962	- 100% accuracy for the Osstem TSIII implants - Osstem USII and 3i Osseotite External were most often confused in the confusion matrix.

If dental records of the implant are missing, it is challenging for a dental clinical to accurately identify implant brand and type, which can hinder effective prosthetic treatment of the patient. Artificial intelligence has great potential to identify and classify implant brands and types, and to bridge experience gaps in dental professionals' performance.

As observed in the other categories as well, CNNs are the preferred ML models when processing radiographic images. Other classifiers, such as SVM, LR or k-NN, do not reach the accuracies obtained by deep learning models like GoogleNet Inception-v3, ResNet or YOLO.

The selected papers collectively emphasize AI’s transformative potential in enhancing implant type identification accuracy, crucial for sound prosthetic restoration decisions. The utilization of varied imaging data sources, such as panoramic and periapical radiographs, forms the basis for AI model training and evaluation. However, a small sample size can limit the ability of AI to accurately identify implants brands and types, highlighting the codependence of automatic solutions and dental practitioners for best results in this medical field.

### Success of Dental Implants

The papers in this category aim to predict implant success probability and risk of periimplantitis. Moayeri et al. [98] applied an ML model which combines results of several classifiers (W-J48, SVM, NN, k-NN) to predict implant success probability, surpassing the accuracy of the best individual classifier. Papantonopoulos et al. [99] used k-means to cluster implants and principal component analysis (PCA) as a variable reduction method for ensemble selection and SVM to predict each implant’s main bone level (IIMBL). Cha et al. [100] and Liu et al. [101] processed various types of radiographic images with convolutional networks in order to detect bone loss around implants. Recent works [102–104] also applied convolutional neural networks on radiographic images (periapical or panoramic) to determine bone loss around implants. Lee et al. [105] evaluated various deep learning architectures in the task of identifying and classifying fractured dental implants.

Ha et al. [106] aimed to identify the most significant factors in predicting the success of dental implants. Wang et al. [107] followed the hypothesis that the probability of periimplantitis can be predicted based on the immune system and applied AI to annotate the tissue-resident immune landscape. Other selected works [108–110] also aimed to predict the risk of periimplantitis based on other data besides radiographs.

Table 4 contains technical details, information about the data used for training/validation/testing, as well as technical and medical results.

**Table 4:** Information extracted from the selected scientific papers in the *success of dental implants* category

References	Year	Aim	Technical details	Data	Results	Medical observations
[98]	2016	- Predict dental implant success probability	- A combined predictive model - Classifiers: W-J48, SVM, NN, k-NN - Combines the four predictive success variable vectors into a stacking learner (Naïve Bayes)	- Parameters from 224 patient cases which had bone graft (no images) - Parameters: gender, age, systemic, smoking, location, placement, loading, diameter, length, system, type, platform, connection, parallel taper, over-denture, sinus lift	- Best single classifier (W-J48): *Acc* 0.893, *Sensitivity* 0.672 - Combined approach: *Acc* 0.902, *Sensitivity* 0.805	- Implant success predictions help medics determine whether an implant is appropriate or not
[99]	2017	- Identify implant phenotypes and predict each implants’ main bone level	- K-means method guided by multidimensional unfolding to cluster implants - PCA and SVM to predict IIMBL - ML classifies peri-implantitis-affected and not-affected implants in a binary fashion	- Radiographic measurements performed by doctor (no images fed to the ML algorithm) – 72 patients with 237 implants - Parameters: age, gender, FMPS, compliance rate with recall schedule, no. of remaining teeth, no. of implants, years of implant function, smoking, diabetes, etc.	Results for predicting IIMBL: - *RMSE*: 0.133 for ES and 0.149 for SVM - 10-fold *cross-validation error*: 0.147 for ES and 0.150 for SVM	–
[106]	2018	- Find the most important factors in predicting implant prognosis	- A decision tree model and SVM to find the most significant factors predicting implant prognosis	- Initial data: 667 implants in 198 patients (no images fed to the ML alg.) - Final data: 59 cases from 53 patients - input: immediate implantation, implant insertion depth, bucco-lingual angulation, medio-distal position and restoration, etc.	- Using decision trees, the mesio-distal position: best *Acc* 0.93 - Using SVM and the LOCCV evaluation method, two sets of only 4 features were sufficient to obtain 0.95 *Acc*: (1) site of placement (SoP), SoP in jaw, posterior SoP, mesio-distal position for restoration; (2) SoP in jaw, implant insertion depth, bucco-lingual angulation, mesio-distal position for restoration.	- When inserting implants, increased attention should be given to locating the precise position of the implant and the mesio-distally distance, to minimize chances of complications and increase success rate.
[105]	2021	- Compare ML methods that identify and classify fractured dental implant type	- Evaluated architectures: VGGNet-19, GoogLeNet Inception-v3 and automated DCNN	- Panoramic/periapical radiographs: from 21398 implants they selected 251 intact and 194 fractured implants + augmentation - 60% for training - 20% for validation - 20% for testing	- Automated DCNN using periapical images—highest detection (*AUC* 0.984) and classification (*AUC* 0.869)	- Periapical images were more appropriate than panoramic ones for the detection and classifying of fractured dental implants
[100]	2021	- Detect bone loss	- 3 separate neural networks that were connected (one that identifies the upper/lower jaws and then one for each jaw) - 1^st^ network: ResNet - The other: Mask R-CNN - Model identifies individual implants and detects keypoints (peri-implant bone level, implant apex, implant top)	- Periapical radiographs: out of 1000 they selected 708 + augmentation - 508 for training - 100 for validation - 100 for testing	- Upper implant detection: *AP* 0.627, *AR* 0.684 - Lower implant detection: *AP* 0.657, *AR* 0.728 - *Mean object keypoint similarity (OKS)*: 0.888 total - *Mean OKS* of dentist: 0.901	- CBCT helps more in detecting and analyzing periimplantitis
[108]	2021	- Predict risk of periimplantitis (binary answer)	- 3 binary classification models: LR, SVM, RF - Evaluated items: history of periodontitis, PCR, smoking, number of occlusal supports by natural teeth, jaw position, fixation method, KMW around each implant	- Radiographic measurements performed by doctor (no images fed to the ML) - From 1408 implants, they selected 254, 127 with and 127 without periimplantitis - 70% for training - 30% for testing	- RF had the highest performance in predicting the onset of periimplantitis: *AUC* 0.71, *Acc* 0.7, *Precision* 0.72, *Recall* 0.66 - SVM: *AUC* 0.64 - LR: *AUC* 0.63	- A correlation between bad smoking habits and severe periodontal diseases
[109]	2021	- Predict the success of dental implants in the presence of imbalanced data	- Imbalanced data is clustered and each cluster is balanced by SMOTE algorithm - The balanced data are classified with 4 methods: decision trees, SVM, kNN, Naïve Bayes - To improve accuracy, an optimal weight is determined for each classifier with a genetic alg.	- Medical data (no images) from 224 patients - Parameters: gender, smoking, implant type, age, implant length, etc. - 70% in each cluster for training - 30% in each cluster for testing	- The hybrid algorithm that contains all classifiers increases the *Acc* by 5.5%, the *Sensitivity* by 0.3% and the *Specificity* by 25%, compared to the best results of a single classifier	- Advantages of bone implants are bone preservation, retention and stability
[107]	2021	- Use AI to render a complete immune atlas of periimplantitis infiltrates	- Fast and robust deconvolution of expression profiles (FARDEEP) – used to employ an RNA-Seq-based approach to annotate the tissue-resident immune landscape - Unsupervised clustering to identify risk groups with distinct immune profiles, microbial colonization dynamics and regenerative outcomes	- Data: PCIF (peri-implant crevicular fluid), no radiographs - Algorithm already trained - testing: 24 patients with at least 1 dental implant diagnosed with periimplantitis - Peri-implant granulation tissues were collected and subjected to RNA-Seq for transcriptome profiling	- They did not evaluate the AI algorithm	- The probability of periimplantitis can be predicted based the immune system - 25% of dental implants are affected by periimplantitis - More M1/M2-like Macrophanges and lower B cell infiltration result in less chances of periimplantitis
[101]	2022	- Detect marginal bone loss around implants	- A Faster R-CNN was trained: Inception Resnet v2	- Periapical radiographs: 1670 images - 1370 for training - 150 for validation - 150 for testing	- For bone loss around implants: *PPV* 0.81, *Sensitivity* 0.67, *Specificity* 0.87 - For lesion sites: *PPV* 0.87, *Sensitivity* 0.75, *Specificity* 0.83 - Agreement between CNN and experts is moderate to substantial (*k* = 0.54 for bone loss sites and 0.568 for bone loss implants)	- A bone resorption of less than 1.5 mm in the first year is acceptable - If 0.2 mm or less is lost each following year it is acceptable
[110]	2023	- Early diagnosis of periimplantitis	- ANN based on bioinformatic analysis: 13 input layers, 5 hidden layers and 2 output layers -13 Hub genes identified with RF classification; - Differentially expressed genes (DEGs) and functional enrichment analyses	- 1380 gene expressions (no images fed to the ML) - The dataset (with the expression profiles) was divided into a training set and a test set (but the number of elements for each set is not mentioned)	- *AUC* of 1 for the test set, indicating a high robustness for periimplantitis diagnosis	- They confirmed an increase in activated NK cells and neutrophils in tissues surrounding periimplantitis - Limitations of their study: small sample size, no images, only periimplantitis transcriptome (no genetic, epigenetic, or environmental factors)
[102]	2023	- Evaluate the degree of periodontal damage around implants	- One CNN (YOLOv2) detects the location of the implant - Image enhancement with histogram equalization - One CNN (AlexNet) assesses the extent of damage caused by periimplantitis	- Periapical radiographs: 456 images - 147 for training the YOLO alg. - 46 for testing the YOLO alg. - 263 images labeled by the YOLO algorithm - After image augmentation: 1304 images for training and 80 images for validating AlexNet	- YOLOv2: *Recall* 0.905, *TNR* 0.78, *Acc* 0.893 - AlexNet: *Acc* 0.904, *Precision* 0.907	- This study represents an assessment of whether periimplantitis has eroded to the first screw thread—a critical indicator of implant stability
[103]	2023	- Predict implant failure	- CNN (ResNet-50) for feature extraction - A hybrid model to combine periapical and panoramic images	- 248 patients, 529 periapical images and 551 panoramic images - Image augmentation = > 900 panoramic & 900 periapical - 5-fold cross-validation: each fold with 720 panoramic & 720 periapical for training and 180 panoramic and 180 periapical for validation	- *AUC* 0.972 for failure with marginal bone loss, 0.947 for failure without marginal bone loss, 0.975 for success - *Acc* 0.87 - *Precision* 0.85 - *Recall* 0.88 - *F1-score* 0.85	- Implant success can be predicted based on radiographic peri-implant alveolar bone pattern (but a better accuracy could be obtained if other patient characteristics would be considered) - A hybrid model that combines periapical and panoramic images can increase accuracy
[104]	2023	- Determine bone loss around implants	- Object detector (YOLOv3) to roughly identify prosthesis (crown) and implant (screw) - Edge detection + edge description to identify significant points (intensity bone changes, intersections between screw and crown)	- 2920 intraoral radiographs - 1460 for training - 1460 for testing	- *mAP* from 0.537 to 0.898	–

AI demonstrates impressive capabilities in predicting implant success probabilities, phenotypes, and bone levels. It excels in identifying factors influencing implant prognosis, emphasizing precise placement.

From Table 4 it can be observed that a popular strategy among the solutions in the periimplantitis category is to not feed the raw data (radiographic images) directly to the ML algorithms, but to use other types of information: either radiographic measurements (determined by doctors), such as implant length, or data extracted from the patient’s medical record, such as smoking habits, age, or gender. The preferred ML models when processing other data besides radiographic images were W-J48, SVM, k-NN, LR, DT, RF or Naïve Bayes. When processing panoramic or periapical radiographs, convolutional networks, such as YOLO, VGGNet-19, GoogLeNet Inception-v3, automated DCNN ResNet, Faster R-CNN or Mask R-CNN were employed.

As can be observed in the other categories as well, each scientific work has its own evaluation metrics: either accuracy, sensitivity, AUC, precision, recall in classifying the implant success probability, or RMSE for predicting each implants’ main bone level, or average precision, recall, PPV in detecting bone loss around implants. Even if the obtained performances presented in the Results column demonstrate AI’s capabilities of predicting periimplantitis risk, detecting fractured dental implants and successfully identifying bone loss around implants, the small sample sizes hint to the need for further, more thorough training and testing procedures.

## Discussions and Conclusion

This paper sets out to identify the most popular ML algorithms applied in the fields of periodontology and implantology, to present technical details and characteristics of data used for training/validation/testing, and to extract interesting medical information.

From the analyzed papers, several observations were drawn:

- When handling radiographic images (either intraoral, panoramic or CBCT datasets), the convolutional neural networks are preferred, since they are able to process raw data with the help of kernels that extract salient characteristics and do not require additional feature extraction steps.
- When processing other data besides radiographic images (for example, when predicting implant success probability based on gender, age, smoking habits, implant placement and other parameters), other ML algorithms, such as SVAM, k-NN, LR, DT, RF or Naïve Bayes are employed.
- For most of the tasks in periodontology and implantology (e.g., predicting periodontally compromised teeth, detecting periodontal bone loss, staging periodontitis, classifying implant brands and types, detecting bone loss around implants), intraoral and panoramic radiographic images provide sufficient information for an accurate result. However, for the task of implant planning (determining the exact location of the implant), CBCT images are preferred.
- Each paper has its own procedure for evaluation, proposing different measurements, such as accuracy, sensitivity, specificity, AUC, RMSE, etc. As presented in “Evaluation Metrics” section, each metric has its own contribution in evaluating the performance of a certain algorithm. Also, depending on the evaluated task (e.g., classification, segmentation), some metrics are applicable while others cannot be used.
- Most of the selected papers have their own datasets which are split into training, validation, and testing. In some cases, for already trained algorithms, the authors provide only a small set for testing. Usually, the sample sizes are small (ranging from tens of images to thousands of images—with only one research work that processed over 150000 radiographs [93]), because of the cumbersome process of manually annotating data. As already stated by Schwendicke et al. [43], a desired scenario would be to shift training sample sizes from several thousands to millions of multi-level connected instances. In a survey by Daneshjou et al. [111] it is mentioned that from a total of 70 analyzed research works and 1 065 291 images which were used to develop or test AI algorithms, only 24.2% were publicly available. Sengupta et al. [112] also addressed the scarcity of publicly available image datasets for machine learning research, claiming that from a total of 332 articles/datasets only one met the selection criteria for oral cancer and was available publicly. Possible reasons for this lack of publicly available datasets are intellectual property protection and commercial benefits. Even though there is a clear trend in the direction of open science, a lot of companies still prefer to protect their investments by limiting the access to source code or labeled data, both being obtained with considerable resources. Another possible reason is the lack of interoperability among imaging and labeling solutions. Actions must be taken world-wide, at government level, to encourage large-scale collaborations between hospitals and e-health providers, to ensure interoperability and standardization in imagining and labeling workflows. For now, data augmentation with simple procedures such as blur, sharpen, color, introduction of noise, translation/rotation, or with complex algorithms such as GAN, can compensate for the limitations of the datasets.

Considering the studied papers, several conclusions are highlighted. Firstly, all the analyzed works underline AI’s role in predicting compromised teeth, staging periodontitis, refining implant predictions, aiding dental decisions and guiding implant suitability. However, there is still room for improvement. The small sample sizes represent an important limitation of the presented solutions. There is a need for publicly available, very large datasets for training, validation, and testing. These would improve the performance of the ML algorithms but would also open the door for the creation of public benchmarks that would allow for more objective evaluations of the proposed solutions. These benchmarks should not only use the same datasets, but the same sets of metrics when evaluating different solutions. Lastly, the aim of these automatic solutions should not be the replacement of doctors, but the assistance offered to medical professionals in all the tasks of periodontology and implantology, to increase both the speed and the quality of the medical act.
