# Correction: Applications of Machine Learning in Periodontology and Implantology: A Comprehensive Review

**DOI:** 10.1007/s10439-024-03565-2

**Published:** 2024-06-21

**Authors:** Cristiana Adina Șalgău, Anca Morar, Andrei Daniel Zgarta, Diana-Larisa Ancuța, Alexandros Rădulea, Ioan Liviu Mitrea, Andrei Ovidiu Tănase

**Affiliations:** 1 University of Agronomic Sciences and Veterinary Medicine of Bucharest, Bucharest, Romania; 2 National University of Science and Technology Politehnica Bucharest, Bucharest, Romania; 3 Minerva University, San Francisco, USA; 4 Cantacuzino National Medical-Military Institute for Research and Development, Bucharest, Romania

**Correction to: Annals of Biomedical Engineering** 10.1007/s10439-024-03559-0

In the original article there is a typographical error in the name of one of the authors. The correct name is Alexandros Rădulea.

